# Evaluation of Hot Workability of Nickel-Based Superalloy Using Activation Energy Map and Processing Maps

**DOI:** 10.3390/ma13163629

**Published:** 2020-08-17

**Authors:** Oleksandr Lypchanskyi, Tomasz Śleboda, Krystian Zyguła, Aneta Łukaszek-Sołek, Marek Wojtaszek

**Affiliations:** Faculty of Metals Engineering and Industrial Computer Science, AGH University of Science and Technology, Av. Mickiewicza 30, 30-059 Krakow, Poland; sleboda@agh.edu.pl (T.Ś.); kzygula@agh.edu.pl (K.Z.); alukasze@metal.agh.edu.pl (A.Ł.-S.); mwojtasz@metal.agh.edu.pl (M.W.)

**Keywords:** nickel-based superalloy, activation energy, processing maps, hot deformation behavior

## Abstract

The stress-strain curves for nickel-based superalloy were obtained from isothermal hot compression tests at a wide range of deformation temperatures and strain rates. The material constants and deformation activation energy of the investigated superalloy were calculated. The accuracy of the constitutive equation describing the hot deformation behavior of this material was confirmed by the correlation coefficient for the linear regression. The distribution of deformation activation energy *Q* as a function of strain rate and temperature for nickel-based superalloy was presented. The processing maps were generated upon the basis of Prasad stability criterion for true strains ranging from 0.2 to 1 at the deformation temperatures range of 900–1150 °C, and strain rates range of 0.01–100 s^−1^. Based on the flow stress curves analysis, deformation activation energy map, and processing maps for different true strains, the undesirable and potentially favorable hot deformation parameters were determined. The microstructural observations confirmed the above optimization results for the hot workability of the investigated superalloy. Besides, the numerical simulation and industrial forging tests were performed in order to verify the obtained results.

## 1. Introduction

The hot deformation of nickel-based superalloys is often associated with the difficulty of selecting optimal parameters of processing. The main difficulties with processing such alloys at low deformation temperatures are connected with excessively high flow stresses while high deformation temperatures are accompanied by rapid grain growth [1]. One such superalloy is Waspaloy, characterized by high resistance to creep fatigue, corrosion resistance, and high strength at high temperatures. It is widely used in a variety of components of gas turbines such as blades, seal rings, and disks working at about 700 °C [2,3,4,5]. Moreover, one of the reasons for the most common use of Waspaloy is its excellent isothermal oxidation resistance [6,7] as well as high relaxation limit at the temperature of 600 °C [8].

The microstructure of Waspaloy consists of γ′ precipitations in the γ matrix. Solution heat treatment and subsequent air cooling promote the formation of primary γ′, while multistage aging promotes precipitation of secondary γ′ particles. Chang et al. [9] noted a noticeable decrease in the strength of this alloy at the temperature of 760 °C because of the solution of γ′ phases and good strength stability at the temperatures not exceeding 649 °C. Yao et al. [10] also indicated insignificant changes in γ′ precipitations in the microstructure of Waspaloy at 650 °C and their partial dissolution with the instability of the secondary γ′ at 700 °C during stress rupture testing. Kearsey et al. [11] noted the dwell fatigue crack growth resistance in Waspaloy and emphasized better damage tolerance over 718 Plus alloy. Zhang M.-C. et al. [4] claimed that the gradual increase of initial grain size improves rupture time in this alloy. The microstructural analysis of precipitation-hardened Waspaloy showed the tendency of an initial unimodal γ′ precipitations size distribution to turn into a bimodal distribution as a result of continued aging [12]. Based on the results of tensile tests, Roy et al. [13] indicated an absence of uniform strain of Waspaloy at the temperatures above 800 °C. The effect of the interrelationship between heat treatment and the forging of Waspaloy was investigated in the work presented by Donachie at al. [14]. It was noted, that the high soaking temperature had a significant effect on the amount of deformation during forging.

In general, the forging temperature limit for Waspaloy is in the range of 1000–1100 °C [15]. The higher forging temperatures can lead to hot shortness and cracking while at lower temperatures inhomogeneous deformation can occur. The γ′ solvus temperature for this alloy is 1030 °C [16]. The authors of work [17] noted, that at strain rates ranging from 0.0001 to 1 s^−1^ dynamic recrystallization (DRX) in this alloy is observed at the temperatures above 1000 °C and low strain rates below 0.01 s^−1^, while dynamic recovery (DRV) occurred at the temperature of 950 °C. Semiatin et al. [18] investigated the recrystallization and plastic flow behavior at supersolvus temperatures of Waspaloy ingot. The successful study of the non-isothermal hot forging of turbine disk made from this alloy was also presented in work [5]. As a result of uniaxial hot compression tests on single crystals of Waspaloy, octahedral slip during alloy deformation was also noted [19].

The study of the behavior of nickel-based superalloys during plastic deformation is essential for the optimization of hot forging processes of such alloys. To characterize the hot deformation behavior and workability of materials, the use of processing maps based upon dynamic material modeling (DMM) was proposed by Prasad et al. [20]. Prasad’s criterion was widely used to describe the hot deformation behavior of nickel-based superalloys [21,22,23,24,25,26,27] and, in particular, Waspaloy [28,29,30]. Amiri et al. [28] developed the processing map for alloy being one of Waspaloy alloy system—AMS 5708 nickel-based superalloy—at the true strain of 0.7 and for a strain rate ranging from 0.01 to 1 s^−1^. The authors of work [29] showed Waspaloy processing map for the strain rate range of 0.01–10 s^−1^, and the true strain of 0.8 s^−1^. Prasad et al. [30] introduced the distribution of the efficiency of power dissipation (*η*) and showed deformation instability regions for Waspaloy also at the true strain of 3.0 s^−1^ for the temperature range of 1010–1093 °C, and strain rates between 0.001 and 0.5 s^−1^. It should be noted that the above studies did not describe the hot-deformation behavior of Waspaloy at high strain rates and various true strains.

Moreover, an important factor is an assessment of the complexity of the hot deformation process, where the crucial role is played by the parameter of deformation activation energy (*Q*). The activation energy *Q* for Waspaloy is noted in the literature [31,32,33] as a constant value at variable temperatures and strain rates. However, it is known that the hot-deformation conditions affect this value.

Taking into account present knowledge, the aim of the present work was focused on characterization of flow behavior of Waspaloy and optimization of hot workability of this type of superalloy at a wide range of temperature and strain rates. Based on the flow stress curves obtained from the isothermal hot compression tests, the processing maps were developed for different true strains for the purpose of better assessment of workability of this material. The distribution of activation energy *Q* at various temperatures, strain rates and true strains allowed much more precise understanding of the Waspaloy deformation mechanism. The verification of the obtained results was performed using the microstructural analysis of the compressed specimens, numerical simulation as well as the drop-forging tests.

## 2. Experimental Procedure

The Waspaloy with chemical composition presented in Table 1 was used in this study. The cylindrical specimens with a diameter of 10 mm and a height of 12 mm were cut out from the Waspaloy rod having a diameter of 50 mm. The isothermal hot-compression tests were performed on Gleeble 3800 thermomechanical (Dynamic Systems Inc., Poestenkill, NY, USA) simulator under vacuum at constant strain rates of 0.01, 0.1, 1, 10, and 100 s^−1^, and at the temperatures of 900, 1000, 1050, 1100 and 1150 °C to a total true strain of 1. Resistance heating of the specimens was carried out up to the processing temperatures at the heating rate of 2.5 °C/s. The samples were held for 10 s before compression for homogenization. A graphite foil was used as a lubricant to reduce friction. After deformation, the samples were quenched in compressed air. Microstructural analysis of the deformed specimens was conducted on LEICA DM4000M optical microscope (Leica Microsystems GmbH, Wetzlar, Germany). The microstructures were observed in a plane parallel to the compression axis through the center of the samples. The specimens for optical observations were etched in etchant consisting of 40 mL HCl, 40 mL C_2_H_5_OH, and 2 g CuCl_2_.

**Figure 1:**
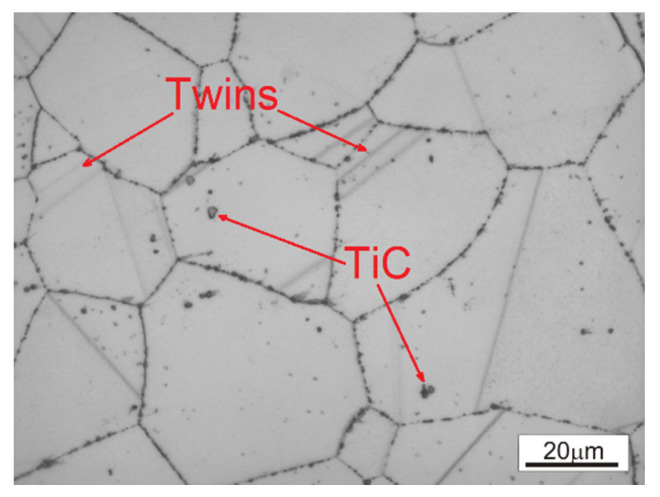
The initial microstructure of Waspaloy.

The starting microstructure of the Waspaloy rod used in this study (Figure 1) is characterized by a relatively homogeneous distribution of equiaxed grains. The precipitations of titanium carbides (TiC) located in the microstructure and finer carbides at the grain boundaries can also be observed. Moreover, annealing twins inside the grains are clearly visible.

Based on the results obtained and the analysis conducted, the numerical simulation of drop forging of gear wheel using QForm 2D/3D commercial software (Qform 2D/3D ver. 8.2.4, QuantorForm Ltd., Moscow, Russia) was performed. During finite element simulation the material behavior, cooling the billet in the air during transportation as well as cooling the tools were taken into account. The boundary conditions included the use of a 10 MN hydraulic screw press with 5 mm/s maximum ram velocity, and the assumed tool temperature of 250 °C. The deformation of the billet of 62 mm in height and 50 mm in diameter consisted of two forging stages.

## 3. Results and Discussion

### 3.1. Flow Stress Curves

**Figure 2:**
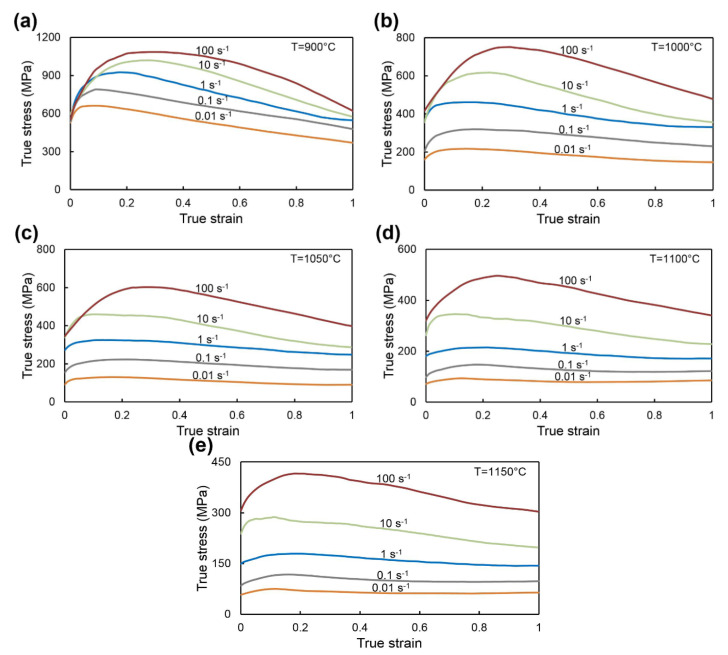
The typical flow stress curves for Waspaloy obtained in compression tests at different strain rates and temperatures: (**a**) 900 °C; (**b**) 1000 °C; (**c**) 1050 °C; (**d**) 1100 °C; (**e**) 1150 °C.

The stress-strain curves of Waspaloy obtained from isothermal hot compression test at different temperatures and strain rates are presented in Figure 2. As expected, the flow stress increases with decreasing deformation temperature and with increasing strain rates. All flow stress curves are characterized by an initial increase in the flow stress until a peak value due to work hardening during deformation. The work-hardening values increased with increasing strain rate or with a decrease in deformation temperature and are directly related to the increase in the dislocation density [32]. The softening behavior of the material can be related to the DRV (dynamic recovery) or dynamic recrystallization (DRX) mechanisms. After reaching peak values at deformation temperatures of 900 and 1000 °C at all strain rates (Figure 2a,b), a constant decrease in flow stress is observed. To a lesser extent, this behavior of the material is observed at 1050 °C for strain rates greater than or equal to 0.1 s^−1^ (Figure 2c) and for the temperatures of 1100, 1150 °C and at high strain rates greater than or equal to 10 s^−1^ (Figure 2d,e). It should be noted that these flow stress curves do not have a stable state and in the case of deformation temperatures of 900 and 1000 °C (Figure 2a,b) such behavior may be related to plastic flow instability during deformation. This instability is most often expressed by the occurrence of shear bands, flow localization, or cracking [29].

The slight softening and subsequent steady state can be noted for flow curves recorded for the temperature of 1050 °C and strain rate of 0.01 s^−1^ (Figure 2c), as well as for flow stress curves obtained at the strain rates smaller than or equal to 1 s^−1^, and for the processing temperatures of 1100 and 1150 °C (Figure 2d,e). Such flow stress curves may indicate the predominant DRX and DRV mechanisms in those processing parameters. It should also be noted that the above analysis is only a part of a comprehensive assessment of the material behavior and further clarification is required.

### 3.2. Constitutive Equation and Activation Energy Map

To assess the deformation difficulty degree, the hot deformation activation energy *Q* must be considered. This physical parameter helps to determine the microstructure evolution and flow-stress behavior during hot deformation as well as providing an opportunity to optimize the hot-working processing parameters. The work hardening and softening mechanisms depend on temperature, strain, and strain rate. To describe the flow behavior of the material and relationships between parameters of deformation temperature, strain rate, and activation energy *Q*, the Arrhenius-type equation is often used [22,34,35]. This constitutive equation in the form of the hyperbolic sine law is universal and successful for use in an extensive stress range and is given as follows:

(1) $$\dot{\varepsilon }=A{\left[\sinh\left(\alpha \sigma \right)\right]}^{n}\exp\left(-\frac{Q}{RT}\right)$$

where: $\dot{\varepsilon }$—strain rate (s^−1^); *T*—deformation temperature (K); *σ*—flow stress, (MPa); *Q*—the deformation activation energy (kJ∙mol^−1^); *R*—the universal gas constant (8.314 J∙mol^−1^∙K^−1^); *α* = *β*/*n*_1_; *A*, *α*, *β*, *n* and *n*_1_—material constants.

The Zener–Hollomon parameter (*Z*) represents the influence of the temperature and strain rate on the deformation behavior, and is expressed as follows:

(2) $$Z=\dot{\varepsilon }\exp\left(\frac{Q}{RT}\right)$$

For all stress levels, the relationship between flow stress and Zener–Hollomon parameter [22] can be written as:

(3) $$Z=A{\left[\sinh\left(\alpha \sigma \right)\right]}^{n}$$

The average values of the materials constants *n*_1_, *β*, and *n* at constant true strain can be calculated as:

(4) $$n_{1}={\left(\frac{\partial \ln\dot{\varepsilon }}{\partial \ln\sigma }\right)}_{T}$$

(5) $$\beta ={\left(\frac{\partial \ln\dot{\varepsilon }}{\partial \sigma }\right)}_{T}$$

(6) $$n={\left[\frac{\partial \ln\dot{\varepsilon }}{\partial \ln(\sinh(\alpha \sigma ))}\right]}_{T}$$

After determining the material constants, the equation for the activation energy of deformation *Q* for all stress levels can be expressed as follows:

(7) $$Q=R\cdot n\cdot \frac{\partial \ln(\sinh(\alpha \sigma ))}{\partial \left(1/T\right)}$$

**Figure 3:**
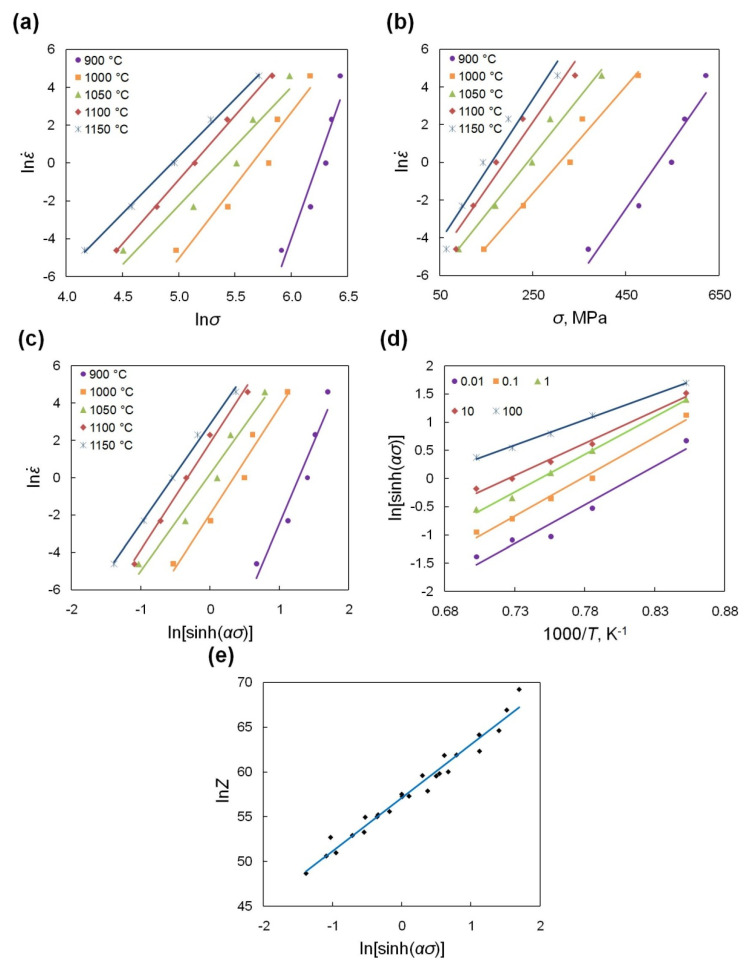
Relationships of (**a**) ln $\dot{\varepsilon }$ vs. ln*σ*, (**b**) ln $\dot{\varepsilon }$ vs. *σ*, (**c**) ln $\dot{\varepsilon }$ vs. ln[sinh(*ασ*)], (**d**) ln[sinh(*ασ*)] vs. (1/*T*) and (**e**) ln*Z* vs. ln[sinh(*ασ*)].

Based on linear regression of the relationships between ln $\dot{\varepsilon }$, ln*σ*, *σ*, and ln[sinh(*ασ*)] (Figure 3a–c) at different deformation temperatures according to Equations (4)–(6), the average values of the material constants *n*_1_, *β*, *α*, and *n* can be determined. The deformation activation energy *Q* for Waspaloy, as a constant value calculated taking into account linear regression of ln[sinh(*ασ*)] − (1/*T*) relation (Figure 3d) at different strain rates based on Equation (7) is 630.17 kJ/mol. Generally, the value of *Q* for different nickel-based superalloys can range from 400 to 1600 kJ/mol [21,36,37,38]. For Waspaloy there is also a discrepancy of this value. Shen et al. [31], McQueen et al. [33], and Matsui [39] calculated the activation energy for the hot working of Waspaloy as equal to 468 kJ/mol, 410 kJ/mol and 384 kJ/mol respectively. Chamanfar et al. [32] calculated the values of *Q* for subsolvus (950–1020 °C) and supersolvus (1060–1140°C) deformation for Waspaloy at strain rates ranging from 0.001 s^−1^ to 1 s^−1^ as 1400 ± 372 kJ/mol and 462 ± 95 kJ/mol, respectively. The calculated value of *Q* for Waspaloy (630.17 kJ/mol) is much larger than the values presented by the authors of works [31,33,39] but fits perfectly in the range indicated by the author of [32]. It should be noted, that this value depends on the investigated deformation conditions ranges such as temperature and strain rate. The activation energy for the hot deformation of Waspaloy is much higher for the self-diffusion activation energy of Ni (278 kJ/mol [40]) or diffusion of Cr in Ni-Cr system (310 kJ/mol [40]). This indicates a possible occurrence of DRX [41,42]. The calculated results of the material constants and the value of *Q* are presented in Table 2.

The variation of the Zener–Hollomon parameter depending on steady flow stress (Figure 3e) is approximately linear and flow stress increases with the increase of the *Z* parameter. The high correlation coefficient for the linear regression (*R*^2^ = 0.997) confirms the accuracy of the constitutive equation (Equation (8)) describing the hot-deformation behavior of Waspaloy.

The constitutive equation of hot deformation for Waspaloy can be expressed as:

(8) $$\dot{\varepsilon }=9.199\times 10^{24}{\left[\sinh\left(0.003856\sigma \right)\right]}^{6.1537}\exp\left(-\frac{630170}{RT}\right)$$

**Figure 4:**
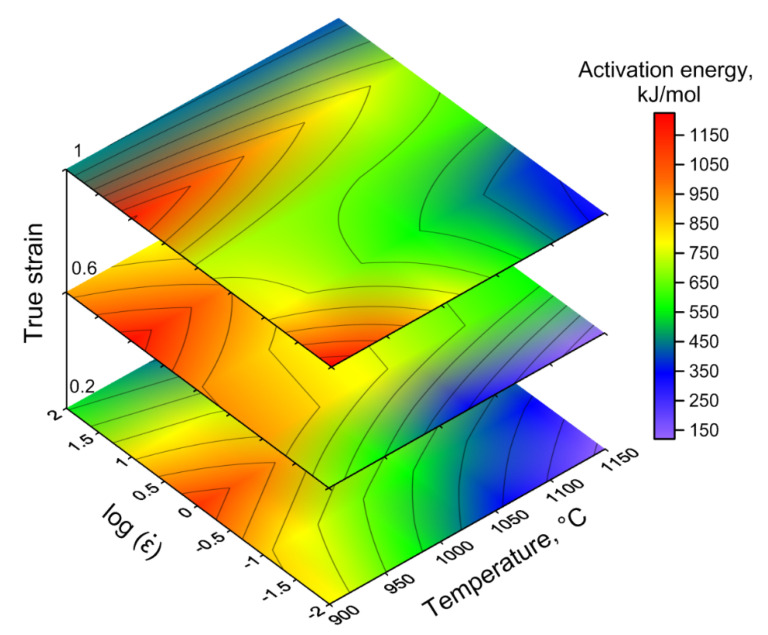
The deformation activation energy map for Waspaloy at true strains of 0.2, 0.6, and 1.

Generally, a constant value of *Q* at various temperatures and strain rates does not take into account the microstructural changes during hot working. On the other hand, it is necessary to know the distribution of *Q* depending on the hot deformation conditions to identify processes associated with DRV, DRX, nucleation, and the growth of precipitation [43]. The distribution of deformation activation energy *Q* as a function of strain rate and temperature for Waspaloy at true strains of 0.2, 0.6, and 1 is presented in Figure 4. The value of *Q* varies in the range from 150 kJ/mol to 1200 kJ/mol during the deformation conditions, which corresponds to the results noted earlier for this superalloy [32], and partially converges with the results obtained for another nickel-based superalloy [44]. In general, there is a noticeable increase in the *Q* parameter with a decrease in the deformation temperature for all true strains, which is due to the increased volume of γ′ precipitations. High deformation temperatures are characterized by the smallest value of activation energy due to the dissolution of γ′ precipitations. Moreover, the increase in the kinetics of dislocation movement, which facilitates deformation, is associated with an increase in temperature (a thermally activated mechanism) [22]. This fact is also the cause of a decrease in the activation energy.

It is considered that DRV is characterized by the deformation activation energy equal or close to the activation energy for self-diffusion. From the distribution of *Q* presented on the activation energy map, and as noted earlier, it can be argued that the DRX is a dominant softening mechanism. It should also be noted that a more constant domain of *Q* value on activation energy map should be considered as optimal for hot working. However, despite the above, for optimization of hot workability of Waspaloy, a comprehensive analysis, including also the processing maps and microstructural observation is needed.

### 3.3. Processing Maps for Waspaloy

The processing maps in accordance with Prasad’s criterion consist of a power dissipation efficiency and flow instability maps. In DMM hot working is strongly connected with power dissipation. One part of this power is dissipated as heat caused by plastic deformation, represented by heat generation (*G*), and the other part, represented by microstructural changes (*J*), is related to the microstructural evolution related to DRV, DRX and phase transformations [20,30]. The total power is evaluated as:

(9) $$P=\sigma \cdot \dot{\varepsilon }=\int_{0}^{\dot{\varepsilon }}\sigma d\dot{\varepsilon }+\int_{0}^{\sigma }\dot{\varepsilon }d\sigma =G+J$$

where: $\dot{\varepsilon }$—strain rate (s^−1^); *σ*—flow stress (MPa).

The strain rate sensitivity parameter (*m*) determines the ratio between heat generation (*G*) and microstructural changes (*J*), and can be determined as [25]:

(10) $$m={\left(\frac{\partial J}{\partial G}\right)}_{T,\varepsilon }={\left(\frac{\partial \log\sigma }{\partial \log\dot{\varepsilon }}\right)}_{T,\varepsilon }$$

where: *T*—temperature (°C); and *ε*—constant true strain value.

The efficiency of power dissipation (*η*) as a function of the strain rate sensitivity can be calculated from:

(11) $$\eta =J/J_{\max}=\frac{2m}{m+1}$$

Based on the results of the distribution of the efficiency of power dissipation *η*, the power dissipation map can be drawn. This map displays the proportion of the power dissipated by the microstructure changes depending on the strain rate and deformation temperature. In general, the higher value of *η* (30–60%) corresponds to DRX, while the lower value (20–30%) is associated with DRV. However, it should be noted that high values of *η* do not always correspond to the safe domains for material workability because the criterion for the occurrence of flow instabilities (*ξ*) should also be taken into account. This instability criterion is given by the following Equation [28,29,30]:

(12) $$\xi (\dot{\varepsilon })=\frac{\partial \log\left(\frac{m}{m+1}\right)}{\partial \log\dot{\varepsilon }}+m\leq 0$$

The variation of *ξ* is represented in a form of instability map as a function of deformation temperature and strain rate. The domains with negative values of this criterion can indicate the occurrence of the flow instability during deformation, such as flow localization, adiabatic shear bands, cracking, and so on [43]. 

**Figure 5:**
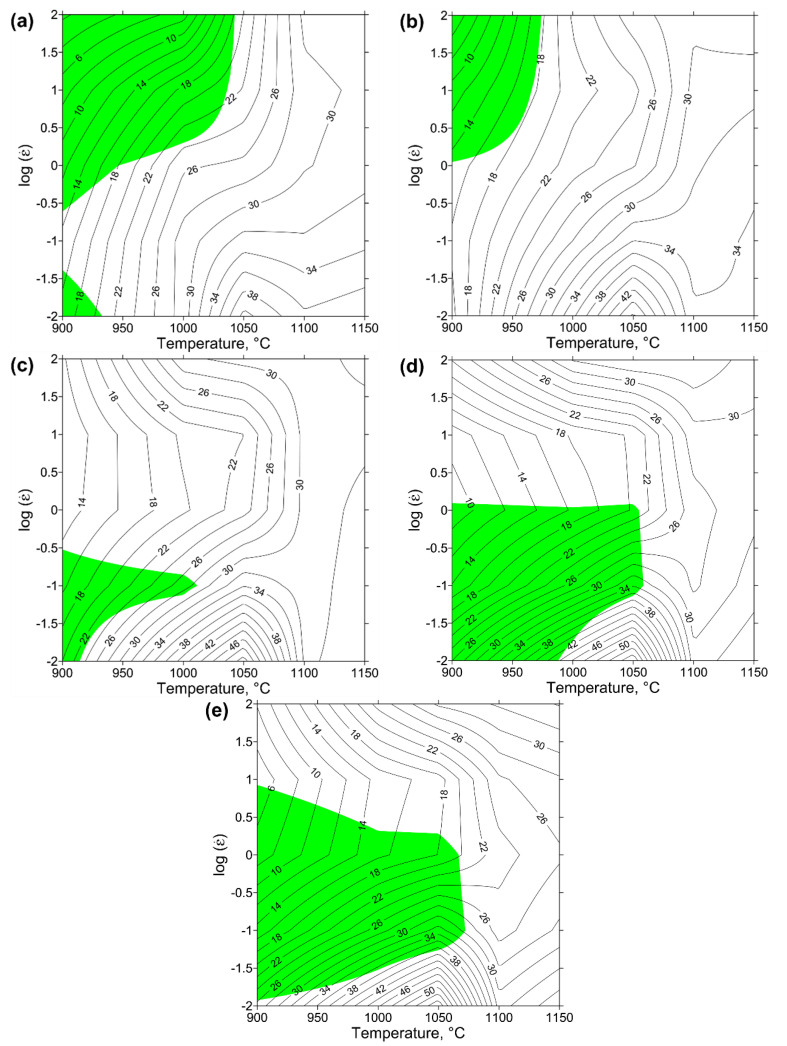
Processing maps for Waspaloy alloy at true strains of (**a**) 0.2, (**b**) 0.4, (**c**) 0.6, (**d**) 0.8 and (**e**) 1.

Figure 5 presents the processing maps for Waspaloy generated upon the basis of the Prasad stability criterion for different true strains (from 0.2 to 1) in a wide range of deformation temperatures (900–1150 °C) and strain rates (0.01–100 s^−1^). These processing maps can be designed by the superimposition of instability maps on power dissipation maps. The black isocline lines describe the distribution of the efficiency of power dissipation (*η*%), while the shaded areas indicate a negative value of instability criterion (*ξ*). Generally, the value of *η* tends to increase with increasing deformation temperature and, to a greater extent, with decreasing strain rate. It can also be noted, that the variation of true strains does not have a significant effect on the distribution of *η*. At all processing maps the domain in which the peak efficiency of power dissipation increases from 40% to 54% with increasing true strain at the temperature of 1050 °C and strain rate 0.01 s^−1^ is observed. This phenomenon can be the result of an increase in the amount of recrystallized grains with increasing true strain, which increases the amount of energy dissipated by ongoing changes in the microstructure. Taking into account that the higher value of this parameter reflects better workability, the hot-working parameters of this domain for the investigated superalloy can be considered as optimal. 

As can be seen from Figure 5, the area of domains covering the potential occurrence of DRX (*η* ≥ 30%) is located mainly at the high temperatures ranging from 1000 to 1150. Moreover, each of the maps shown in Figure 5 is characterized by isocline curvature changes near the temperature of 1050 °C. This effect can be associated with the γ′ solvus of Waspaloy [16] near this temperature. The same results of the energy dissipated during dissolution and phase transformation were noted for other nickel-based superalloys [45].

The instability domains with negative vales of *ξ* (shaded areas) presented in Figure 5a–c represent the undesirable hot deformation conditions for Waspaloy due to the possibility of the occurrence of flow instability. As can be seen from these figures, the location of the instability regions changes with the true strain value at the temperatures ranging from 900 °C to 1050 °C at the whole range of the investigated strain rates. This fact indicates, that the state of strain at the temperatures below 1050 °C is unstable.

The processing map for the true strain of 0.4 (Figure 5b) indicates the behavior of Waspaloy at the flow softening state. Two instability domains on the processing map, describing the behavior of the material at a true strain of 0.2 (Figure 5a), can be distinguished. The largest domain is located at the temperature range of 900–1040 °C and the strain rates of 0.3–100 s^−1^, and decreases with increasing true strain (Figure 5b), and cannot be noticed at true strains larger or equal to 0.6 (Figure 5c–e). On the other hand, the smaller instability domain (Figure 5a), limited by temperatures ranging from 900 °C to 935 °C and low strain rates (0.01–0.04 s^−1^), is decreasing with increasing values of true strain to 0.4 (Figure 5b). However, with a further increase of true strain to the values larger or equal to 0.6, this domain tends to increase the area of instability, which is clearly visible in Figure 5c–e. Generally, the location of instability domain for Waspaloy shown in Figure 5c, at a true strain of 0.8, corresponds to the results presented in previous works [28,29] for the same conditions as well as showing a good match for the higher value of true strain [30]. However, it can be noted that the developed processing maps (Figure 5) provide a better understanding of the behavior of the material in a wider range of temperatures, strains, and strain rates.

It should also be noted that to assess the hot workability of the material based on processing maps it is necessary to take into account the hot deformation behavior at each true strain level. This is primarily due to the complexity of the geometry of forged products, which affects the values of true strains [46]. Complex analysis of the processing maps (Figure 5) as well as the analysis of the deformation activation energy map (Figure 4) and stress-strain curves (Figure 2) for Waspaloy, allows describing the undesirable and potentially favorable areas of hot deformation parameters. The undesirable areas include relatively low processing temperatures (from 900 °C to 1000 °C) and low strain rate range (0.01–0.04 s^−1^) combined with a processing temperature range of 900–1050 °C and strain rates ranging from 0.04 s^−1^ to 100 s^−1^. They are characterized by the lowest efficiency of power dissipation (Figure 5a–c), which means the worst workability and highest *Q* values (Figure 4). Moreover, the analysis of flow stress curves (Figure 2) and instability domains presented in Figure 5a–c indicates the presence of plastic flow instabilities that may occur during deformation. The optimal hot deformation conditions for Waspaloy can be described as processing at temperatures from 1100 °C to 1150 °C and at all investigated strain rates as well as the area at the temperature range of 1025–1100 °C and strain rates ranging from 0.01 to 0.04 s^−1^. The advantages of these processing conditions indicated on the processing maps include the low (equal or greater than 750 kJ/mol) deformation activation energy values (Figure 4) and high (equal or greater than 24%) *η* values (Figure 5a–c), which combined with the flow stress curves analysis indicate the occurrence of DRX as dominant mechanism. In addition, these processing maps areas do not contain the instability domains and are located far from them. It should be emphasized, that the successful non-isothermal hot forging of turbine disk was carried out under the conditions relevant to these processing maps areas [5]. However, the above analysis needs to be confirmed by the microstructure observations of the compressed specimens.

### 3.4. Microstructural Observations

**Figure 6:**
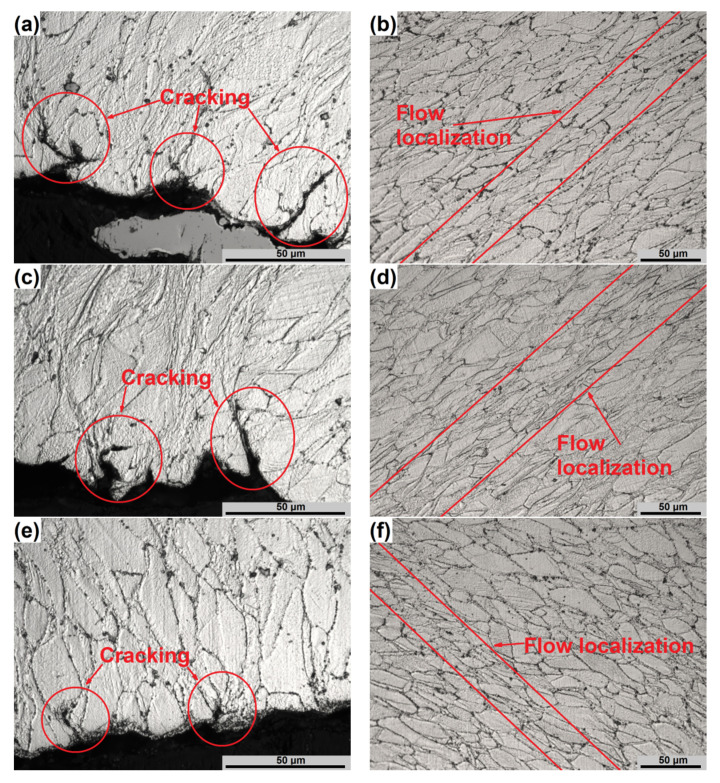
Typical microstructures of the specimens deformed at the temperatures and strain rates of (**a**,**b**) 900 °C/0.1 s^−1^, (**c**,**d**) 900 °C/1 s^−1^, (**e**,**f**) 1000 °C/0.1 s^−1^ at instability domain.

The microstructures of the specimens obtained in the instability conditions are presented in Figure 6. In the instability domain, the cracking on the surface (Figure 6a,c,e) and localized plastic flow (Figure 6b,d,f) were observed. In all cases, a pronounced inhomogeneity of deformation can be observed. Cracking of the compressed specimens, noted on the surface at the areas of the occurrence of shear cracks [29], occurred along the grain boundaries. One of the reasons for their occurrence is flow localization (Figure 6b,d,f) located near these cracks. The presence of γ′ precipitations restricts the movement of grain boundaries, which is also reflected and proven by high *Q* value and potential low workability under these conditions. At low temperatures (Figure 6a–d), the occurrence of the processes characteristic for DRX should not be considered. At higher temperatures (Figure 6e,f), partial DRX and dissolution of γ′ precipitations can be observed, which reduces the intensity of cracking due to flow localization. It confirms the analysis of the processing maps.

Moreover, as can be seen from Figure 6b,d,f, flow localization has a form of local deformation bands oriented at an angle of 45° to the compression direction. Further deformation under indicated instabilities can lead to adiabatic shear bands and, subsequently, to shear cracks. Considering the above, it can be concluded, that the hot deformation of Waspaloy in undesirable areas is not recommended.

**Figure 7:**
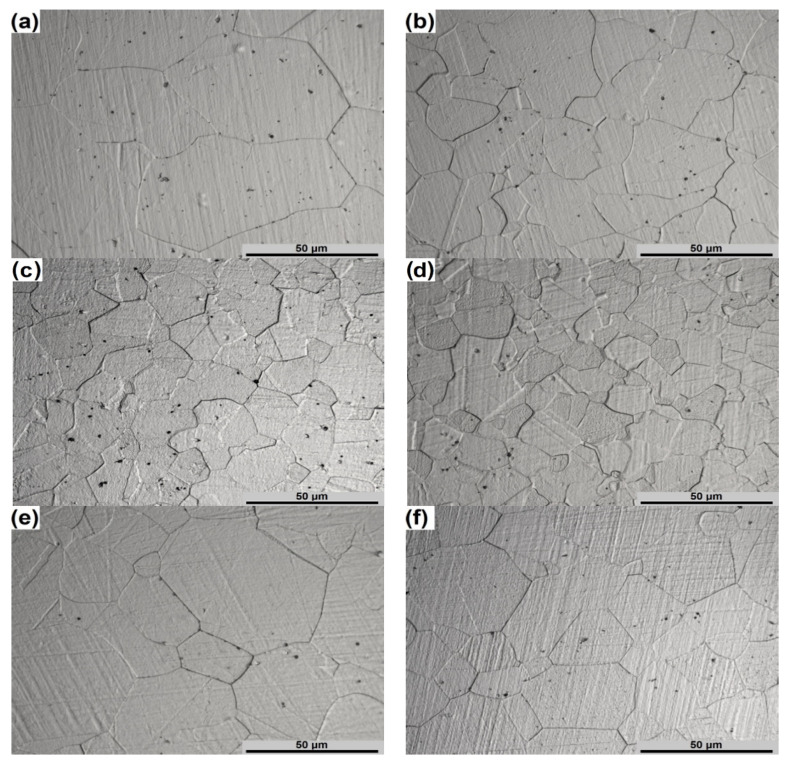
The microstructures of the specimens deformed under the following processing conditions: (**a**) 1050 °C/0.01 s^−1^; (**b**) 1100 °C/0.1 s^−1^; (**c**) 1100 °C/1 s^−1^; (**d**) 1100 °C/100 s^−1^; (**e**) 1150 °C/0.1 s^−1^ and (**f**) 1150 °C/100 s^−1^.

Figure 7 presents the microstructures of the specimens compressed under optimal hot deformation conditions. As can be seen from this figure, the DRX mechanism is more or less characteristic for each of the deformation conditions presented. The low strain rate (0.01 s^−1^) leads to an increase in the exposure of the material to a given temperature, resulting in a large part of γ′ precipitations and carbides as well as in recrystallized equiaxed grain growth (Figure 7a). An increase of deformation temperature will lead to even greater growth of grains while decreasing the temperature will increase the volume fraction of γ′ particles, which will reduce the workability of the investigated superalloy. It also should be noted, that the presence of carbides and γ′ particles inhibit grain growth [32]. Taking into account these facts as well as the previous analysis, it can be argued that these deformation conditions are optimal at strain rates ranging from 0.01 to 0.04 s^−1^. Higher strain rates (0.04–100 s^−1^) will reduce the exposure time of the material to deformation temperature and, therefore, will increase the temperature of dissolution of large volume fraction of γ′ precipitations.

The microstructures presented in Figure 7b–f confirm the expediency of hot deformation of Waspaloy at temperatures ranging from 1100 °C to 1150 °C and strain rate range of 0.04–100 s^−1^. These microstructures are characterized by almost full DRX and good stability of the microstructures. As can be seen from Figure 7b–d, the hot deformation at the temperature of 1100 °C will provide a finer grain size.

### 3.5. The Drop-Forging Test in Industrial Conditions

Based on the results obtained, relating to hot deformation conditions for the investigated superalloy, the numerical simulation as well as the drop forging test of the selected part (gear wheel) in industrial conditions was performed. Generally, it was found that the stable process of hot working of the investigated alloy took place in the ranges of high deformation temperatures (≥1050 °C). Moreover, high deformation temperatures are characterized by lower values of deformation activation energy (Figure 4). Due to the above and taking into account the temperature drop during deformation, the billet temperature was selected as 1150 °C.

**Figure 8:**
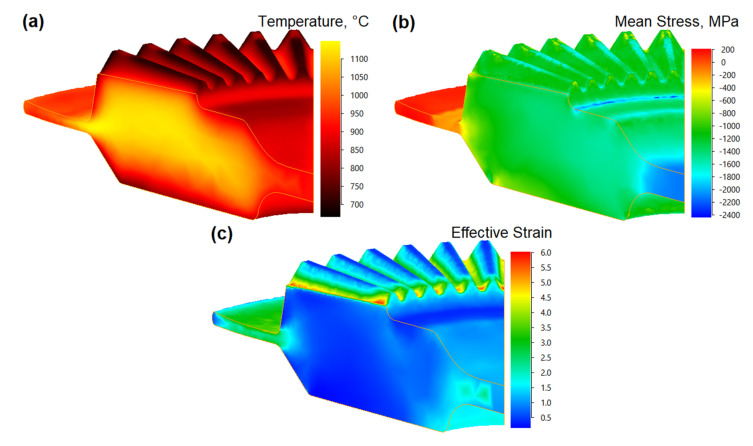
The results obtained for the temperature distribution: (**a**), mean stress (**b**), and effective strain (**c**) in the drop-forged Waspaloy gear wheel.

The numerical distributions of temperature, mean stress and effective strain in the forged gear wheel in a final stage are shown in Figure 8. Figure 8a shows that the temperature in the forged part was within the range from 1050 °C to 1150 °C which corresponded to the favorable hot deformation conditions. The decrease in the temperature was recorded at the contact of the workpiece with the tool while the highest temperature values were observed in the flash zones. Almost the entire volume of the forging had a similar mean stress distribution (Figure 8b). The only exceptions were the areas of the flash and web regions where the largest values of mean stress could be observed, however, these areas might be disregarded due to destined for being cut off. The distribution of the effective strain (Figure 8c) indicated an intensive material flow in the area of gear wheel teeth and flash.

**Figure 9:**
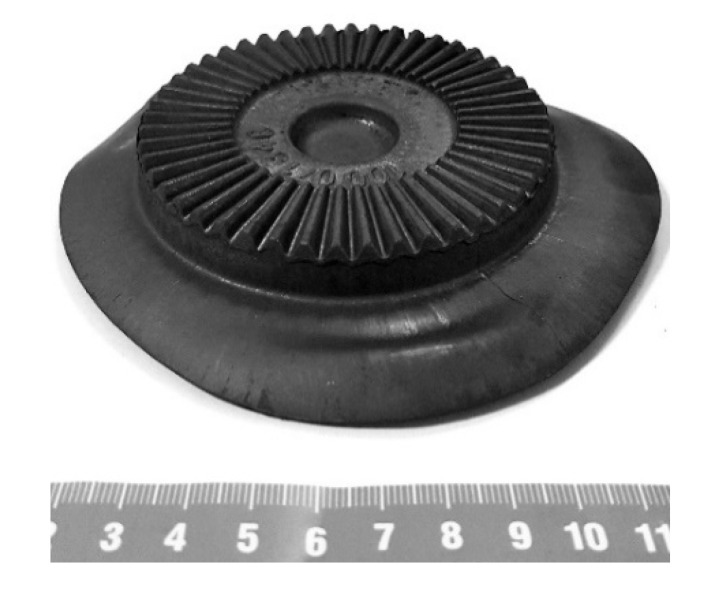
Drop forged gear wheel.

The verification based on a complex analysis of the previously obtained results was carried out by industrial drop forging tests of a gear wheel (Figure 9) performed on the 10 MN hydraulic screw press.

**Figure 10:**
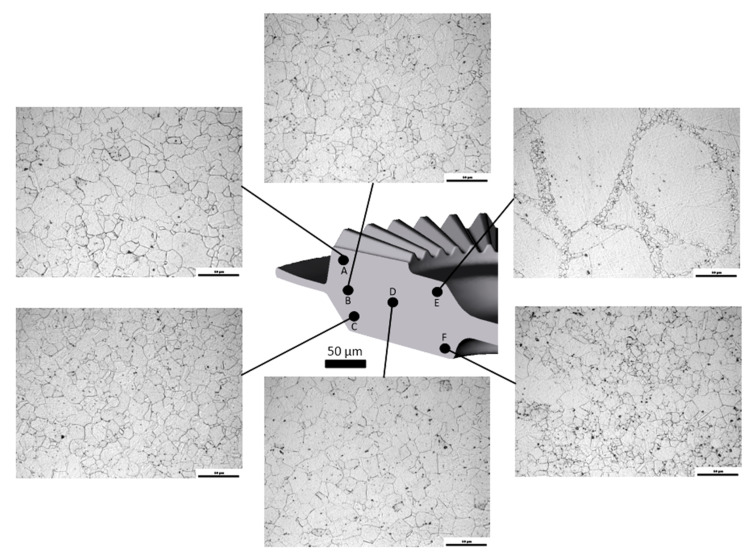
The microstructure of the Waspaloy forged part observed at various areas of its cross-section (areas marked from **A** to **F**).

After drop-forging tests, the quality of the obtained gear wheel was evaluated. As can be seen in Figure 10 describing the microstructure of the forged part is defined as characteristic areas, the microstructure had no visible defects. The microstructure in the investigated areas showed the occurrence of the DRX process, which resulted in a partial or complete replacement of the initial microstructure by uniform grains. Also, the beginning of DRX at the initial grains boundaries in the form of fine recrystallization nuclei can be seen in the area E (Figure 10). Almost full DRX can be observed at the areas A, B, C, and D. Generally, basing on the presented microstructures it can be concluded, that the obtained forged part had a good quality that confirmed the above results concerning the optimization of hot-processing parameters for Waspaloy.

## 4. Conclusions

The stress-strain curves for Waspaloy were obtained from isothermal hot compression tests performed to a total true strain of 1 at strain rates ranging from 0.01 s^−1^ to 100 s^−1^, and at temperatures in the range of 900–1150 °C. Based on these tests, calculations, and microstructural observations, the following conclusions can be summarized:

- The average activation energy (*Q*) value, calculated for the investigated Waspaloy, was 630.17 kJ/mol. The high correlation coefficient for the linear regression *R*^2^ = 0.997 confirmed the accuracy of the constitutive equation describing the hot-deformation behavior of the investigated superalloy. The distribution of activation energy as a function of strain rate and temperature for Waspaloy at different strain rates varied in the range from 150 kJ/mol to 1200 kJ/mol.
- The undesirable processing conditions include low temperatures (from 900 °C to 1000 °C) and low strain rates range (0.01–0.04 s^−1^) combined with the processing temperature range of 900–1050 °C, and strain rates ranging from 0.04 s^−1^ to 100 s^−1^.
- The most favorable hot-deformation conditions for Waspaloy can be described by the processing map area for the temperatures from 1100 °C to 1150 °C, and at all investigated strain rates as well as the area for the temperatures range of 1025–1100 °C, and strain rates ranging from 0.01 to 0.04 s^−1^.
- The microstructures of the specimens obtained in the instability conditions confirmed the occurrence of the flow instability during deformation. The microstructures of the specimens compressed at optimal hot deformation conditions were characterized by almost full DRX and good deformation stability.
- The numerical analyses as well as drop forging tests of a gear wheel confirmed the results related to optimization of hot-forging parameters for Waspaloy.

## Figures and Tables

**Table 1:** Chemical composition of Waspaloy alloy used in this study (wt.%).

Cr	Co	Mo	Ti	Al	Fe	Zr	Mn	Nb	W	Si	C	V	Cu	Ni
19.48	13.25	4.33	3.08	1.35	0.93	0.06	0.05	0.04	0.04	0.04	0.033	0.03	0.01	Bal.

**Table 2:** Calculated material constants and deformation activation energy for Waspaloy.

*n*_1_	*β*	*α*	*n*	*A*	*Q*, kJ/mol
8.74	0.0337	0.003856	6.1537	9.199 × 10^24^	630.170
